# Supplementary material for: The familial risk of infection-related hospitalization in children: A population-based sibling study
Source: PLoS One. 2021 Apr 28;16(4):e0250181. doi: 10.1371/journal.pone.0250181 (PMC8081236; doi:10.1371/journal.pone.0250181)
Supplement: S1 Fig — Infection-related hospitalization sibling hazard ratios among probands whose sibling/s had infection-related hospitalization/s for the same specific infection compared to risk in probands whose siblings/s did not have infection-related hospitalizations (reference group), among younger and older siblings. Adjusted for maternal age, parity, gestational age, birth weight, sex, season of birth, 5-minute Apgar score, birth mode, and birth year. ICD 9 and 10 codes for bronchiolitis: 466.1, J21; gastroenteritis: 009, 535.4 535.7, A09; otitis media chronic: 381.1, 381.2, 381.3, 381.4, 382.1, 382.2, 382.3, H65.2, H65.3, H65.4, H65.9, H66.1, H66.2, H66.3; tonsillitis acute: 034.0, 463, J03; tonsillitis chronic: 474, J35. Acute otitis media 381.0, 382.0, H65.0, H65.1, H66.0 was uncommon and not included. (PDF) [file pone.0250181.s001.pdf]

S1 Fig. Infection-related hospitalization sibling hazard ratios for specific infection groups
Infection-related hospitalization sibling hazard ratios among probands whose sibling/s had infection-related hospitalization/s for the same specific infection compared to risk in probands whose siblings/s did not have infection-related hospitalizations (reference group), among younger and older siblings

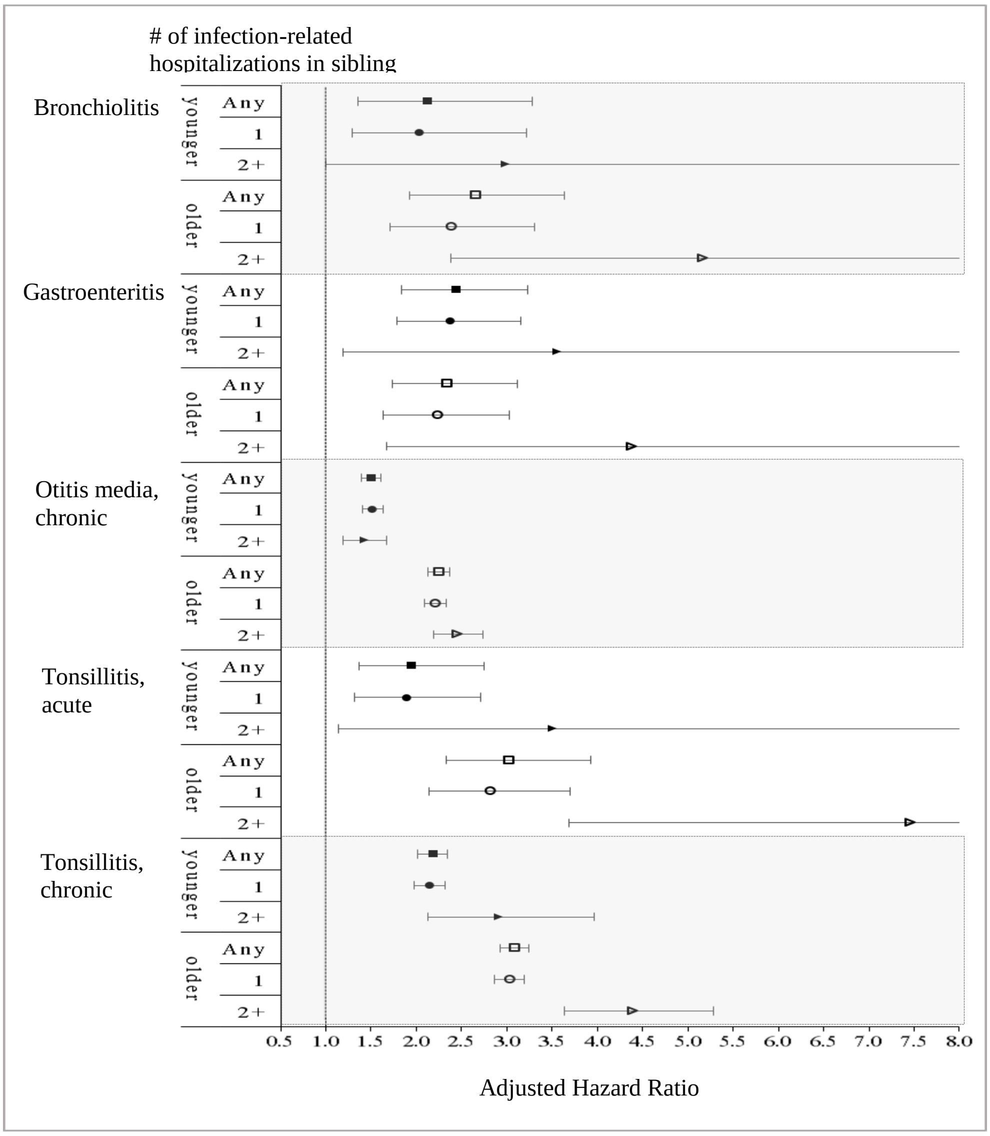

Adjusted for maternal age, parity, gestational age, birth weight, sex of the child, season of birth, 5-minute Apgar score, birth mode, birth year.

ICD 9 and 10 codes for bronchiolitis: 466.1, J21; gastroenteritis: 009, 535.4 535.7, A09; otitis media chronic: 381.1, 381.2, 381.3, 381.4, 382.1, 382.2, 382.3, H65.2, H65.3, H65.4, H65.9, H66.1, H66.2, H66.3; tonsillitis acute: 034.0, 463, J03; tonsillitis chronic: 474, J35. Acute otitis media 381.0, 382.0, H65.0, H65.1, H66.0 was uncommon and not included.
