# Supplementary material for: The familial risk of infection-related hospitalization in children: A population-based sibling study
Source: PLoS One. 2021 Apr 28;16(4):e0250181. doi: 10.1371/journal.pone.0250181 (PMC8081236; doi:10.1371/journal.pone.0250181)

S2 Fig. Time from exposure (sibling infection-related hospitalization) to outcome (proband infection-related hospitalization) for all and specific infection groups

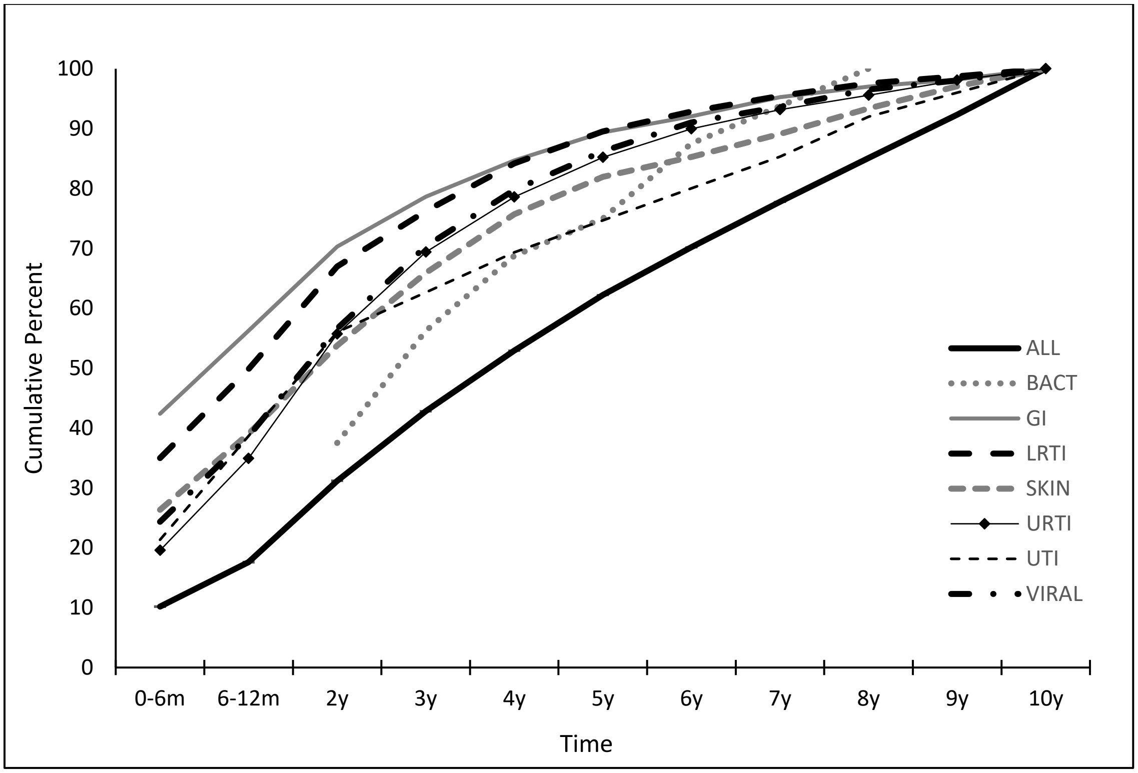

Supplement: S2 Fig — (PDF) [file pone.0250181.s002.pdf]
