# Supplementary material for: The familial risk of infection-related hospitalization in children: A population-based sibling study
Source: PLoS One. 2021 Apr 28;16(4):e0250181. doi: 10.1371/journal.pone.0250181 (PMC8081236; doi:10.1371/journal.pone.0250181)
Supplement: S1 Table — (PDF) [file pone.0250181.s003.pdf]

S1 Table. Sensitivity analysis of infection-related hospitalization identification

MODEL	# of sibling infection-related hospitalizations	Infection-related hospitalizations identified by: (Original analysis) Principal + up to 20 additional diagnostic codes	Principal + 3 additional diagnostic codes
Crude	1	1.40 (1.38-1.41)	1.40 (1.38-1.42)
	2	1.61 (1.57-1.65)	1.60 (1.57-1.64)
	3+	1.75 (1.69-1.81)	1.68 (1.62-1.74)
Adjusted	1	1.41 (1.39-1.43)	1.41 (1.39-1.42)
	2	1.65 (1.61-1.69)	1.64 (1.60-1.68)
	3+	1.83 (1.77-1.90)	1.74 (1.68-1.81)
Adjusted + SEIFA	1	1.39 (1.37-1.41)	1.41 (1.39-1.42)
	2	1.55 (1.51-1.58)	1.63 (1.59-1.67)
	3+	1.56 (1.51-1.62)	1.73 (1.67-1.80)
Adjusted + smoking	1	1.42 (1.40-1.45)	1.45 (1.42-1.48)
	2	1.60 (1.55-1.66)	1.69 (1.62-1.75)
	3+	1.74 (1.65-1.83)	1.86 (1.75-1.97)
Adjusted + SEIFA + smoking	1	1.42 (1.40-1.45)	1.45 (1.42-1.48)
	2	1.60 (1.54-1.66)	1.69 (1.62-1.76)
	3+	1.76 (1.67-1.87)	1.87 (1.76-1.99)
Adjusted 1 sibling	1	1.47 (1.45-1.50)	1.47 (1.45-1.50)
	2	1.72 (1.67-1.78)	1.73 (1.67-1.79)
	3+	1.81 (1.73-1.89)	1.81 (1.73-1.89)
Adjusted 2 siblings	1	1.23 (1.21-1.26)	1.25 (1.22-1.28)
	2	1.48 (1.42-1.54)	1.47 (1.41-1.53)
	3+	1.61 (1.5-1.71)	1.68 (1.56-1.79)
Adjusted 3 siblings	1	1.26 (1.22-1.3)	1.24 (1.19-1.28)
	2	1.34 (1.28-1.41)	1.38 (1.30-1.46)
	3+	1.46 (1.35-1.57)	1.44 (1.30-1.58)

All younger siblings	1	1.34 (1.31-1.37)	1.33 (1.30-1.36)
	2	1.54 (1.49-1.6)	1.50 (1.45-1.56)
	3+	1.65 (1.58-1.73)	1.61 (1.54-1.70)
All older siblings	1	1.47 (1.44-1.5)	1.49 (1.46-1.52)
	2	1.72 (1.66-1.79)	1.72 (1.65-1.79)
	3+	1.74 (1.63-1.85)	1.82 (1.71-1.94)
