# Supplementary material for: The familial risk of infection-related hospitalization in children: A population-based sibling study
Source: PLoS One. 2021 Apr 28;16(4):e0250181. doi: 10.1371/journal.pone.0250181 (PMC8081236; doi:10.1371/journal.pone.0250181)
Supplement: Attachment — Submitted filename: PONE-D-20-33021_reviewer_AS.pdf [file pone.0250181.s005.pdf]

PLOS ONE

The familial risk of infection-related hospitalization: a population-based sibling study

--Manuscript Draft--

**Manuscript Number:**	PONE-D-20-33021
**Article Type:**	Research Article
**Full Title:**	The familial risk of infection-related hospitalization: a population-based sibling study
**Short Title:**	Sibling risk of infection-related hospitalization
**Corresponding Author:**	Jessica E. Miller, Ph.D<br>Murdoch Children's Research Institute<br>Parkville, Victoria AUSTRALIA
**Keywords:**	siblings; childhood infection; infection-related hospitalization; pediatrics
**Abstract:**	Objective: To assess the risk of severe childhood infections within families, we conducted a sibling analysis in a population-based cohort study with genealogical linkage. We investigated the sibling risk of hospitalization with common infections, a marker of severity. We hypothesized that having siblings hospitalized for infection would increase the proband's subsequent risk of admission with infection.<br><br>Study design: We used population data on Western Australian live-born singletons and their siblings between 1980 and 2014. Measures of infection were infection-related hospitalizations from discharge diagnostic codes. Exposure was having a sibling who had an infection-related hospitalization. Outcomes were infection-related hospitalizations in the child/proband. Probands were followed until an infection-related hospitalization admission (up to the first three), death, 18th birthday, or end of 2014, whichever occurred first. Infection risks were estimated by adjusted Cox proportional hazard models for multiple events.<br><br>Results: Of 512,279 probands, 142,915 (27.9%) had infection-related hospitalizations; 133,322 (26.0%) had a sibling with a previous infection-related hospitalization (i.e. exposed). Median interval between sibling and proband infection-related hospitalizations was 1.4 years (inter-quartile range 0.5-3.7). Probands had a dose-dependent increase in risk if sibling/s had 1, 2, or 3+ infection-related hospitalizations (adjusted hazard ratio, aHR 1.41, 95% CI 1.39-1.43; aHR 1.65, 1.61-1.69; aHR 1.83, 1.77-1.90, respectively). Among siblings with the same clinical infection type, highest sibling risks were for genitourinary (aHR 2.06, 1.68-2.53), gastrointestinal (aHR 2.07, 1.94-2.19), and skin/soft tissue infections (aHR 2.34, 2.15-2.54). Overall risk of infection-related hospitalization was higher in children with more siblings and with older siblings.<br><br>Conclusion: In this population-based study, we observed an increased risk of infection-related hospitalization in children whose siblings were previously hospitalized for infection. Public health interventions may be particularly relevant in families of children hospitalized with infection.
**Order of Authors:**	Jessica E. Miller, Ph.D
	Kim W. Carter
	Nicholas de Klerk
	David P. Burgner
**Additional Information:**	
**Question**	**Response**
**Financial Disclosure**<br><br>Enter a financial disclosure statement that describes the sources of funding for the work included in this submission. Review	This work is supported by the Australian National Health and Medical Research Council GTN572742, GTN106594, and GTN1064829 and GTN1175744 to DPB. Research at Murdoch Children's Research Institute is supported by by the Victorian Government's Operational Infrastructure Support Program.<br>The funders had no role in study design, data collection and analysis, decision to

Powered by Editorial Manager® and ProduXion Manager® from Aries Systems Corporation

the submission guidelines for detailed requirements. View published research articles from *PLOS ONE* for specific examples.<br><br>This statement is required for submission and **will appear in the published article** if the submission is accepted. Please make sure it is accurate.<br><br>**Unfunded studies**<br>Enter: *The author(s) received no specific funding for this work.*<br><br>**Funded studies**<br>Enter a statement with the following details:<br>• Initials of the authors who received each award<br>• Grant numbers awarded to each author<br>• The full name of each funder<br>• URL of each funder website<br>• Did the sponsors or funders play any role in the study design, data collection and analysis, decision to publish, or preparation of the manuscript?<br>• **NO** - Include this sentence at the end of your statement: *The funders had no role in study design, data collection and analysis, decision to publish, or preparation of the manuscript.*<br>• **YES** - Specify the role(s) played.<br><br>* typeset	publish, or preparation of the manuscript.
**Competing Interests**<br><br>Use the instructions below to enter a competing interest statement for this submission. On behalf of all authors, disclose any competing interests that could be perceived to bias this work—acknowledging all financial support and any other relevant financial or non-financial competing interests.<br><br>This statement **will appear in the published article** if the submission is accepted. Please make sure it is accurate. View published research articles from *PLOS ONE* for specific examples.	The authors have declared that no competing interests exist.

**NO authors have competing interests**<br><br>Enter: *The authors have declared that no competing interests exist.*<br><br>**Authors with competing interests**<br><br>Enter competing interest details beginning with this statement:<br><br>*I have read the journal's policy and the authors of this manuscript have the following competing interests: [insert competing interests here]*<br><br>* typeset	
**Ethics Statement**<br><br>Enter an ethics statement for this submission. This statement is required if the study involved:<br><br>• Human participants<br>• Human specimens or tissue<br>• Vertebrate animals or cephalopods<br>• Vertebrate embryos or tissues<br>• Field research<br><br>Write "N/A" if the submission does not require an ethics statement.<br><br>General guidance is provided below. Consult the submission guidelines for detailed instructions. **Make sure that all information entered here is included in the Methods section of the manuscript.**	The study was approved by the Department of Health Human Research Ethics Committee (HREC), Murdoch Children's Research HREC and University of WA HREC.

Powered by Editorial Manager® and ProduXion Manager® from Aries Systems Corporation

**Format for specific study types**<br><br>**Human Subject Research (involving human participants and/or tissue)**<br>• Give the name of the institutional review board or ethics committee that approved the study<br>• Include the approval number and/or a statement indicating approval of this research<br>• Indicate the form of consent obtained (written/oral) or the reason that consent was not obtained (e.g. the data were analyzed anonymously)<br><br>**Animal Research (involving vertebrate animals, embryos or tissues)**<br>• Provide the name of the Institutional Animal Care and Use Committee (IACUC) or other relevant ethics board that reviewed the study protocol, and indicate whether they approved this research or granted a formal waiver of ethical approval<br>• Include an approval number if one was obtained<br>• If the study involved *non-human primates*, add *additional details* about animal welfare and steps taken to ameliorate suffering<br>• If anesthesia, euthanasia, or any kind of animal sacrifice is part of the study, include briefly which substances and/or methods were applied<br><br>**Field Research**<br><br>Include the following details if this study involves the collection of plant, animal, or other materials from a natural setting:<br>• Field permit number<br>• Name of the institution or relevant body that granted permission	
**Data Availability**<br><br>Authors are required to make all data underlying the findings described fully available, without restriction, and from the time of publication. PLOS allows rare exceptions to address legal and ethical concerns. See the PLOS Data Policy and FAQ for detailed information.	No - some restrictions will apply

A Data Availability Statement describing where the data can be found is required at submission. Your answers to this question constitute the Data Availability Statement and **will be published in the article**, if accepted.<br><br>**Important:** Stating 'data available on request from the author' is not sufficient. If your data are only available upon request, select 'No' for the first question and explain your exceptional situation in the text box.<br><br>Do the authors confirm that all data underlying the findings described in their manuscript are fully available without restriction?	
**Describe where the data may be found in full sentences. If you are copying our sample text, replace any instances of XXX with the appropriate details.**<br><br>• If the data are **held or will be held in a public repository**, include URLs, accession numbers or DOIs. If this information will only be available after acceptance, indicate this by ticking the box below. For example: *All XXX files are available from the XXX database (accession number(s) XXX, XXX.).*<br>• If the data are all contained **within the manuscript and/or Supporting Information files**, enter the following: *All relevant data are within the manuscript and its Supporting Information files.*<br>• If neither of these applies but you are able to provide **details of access elsewhere**, with or without limitations, please do so. For example:<br><br>*Data cannot be shared publicly because of [XXX]. Data are available from the XXX Institutional Data Access / Ethics Committee (contact via XXX) for researchers who meet the criteria for access to confidential data.*<br><br>*The data underlying the results presented in the study are available from (include the name of the third party*	Please visit the Data Linkage Western Australia website: https://www.datalinkage-wa.org.au/<br>for information regarding access to similar data used in this project.

and contact information or URL).<br>• This text is appropriate if the data are owned by a third party and authors do not have permission to share the data.<br><br>* typeset	
Additional data availability information:	

Powered by Editorial Manager® and ProduXion Manager® from Aries Systems Corporation

Manuscript

Click here to access/download;Manuscript;Sibling manuscript 14Oct2020.docx

The familial risk of infection-related hospitalization: a population-based sibling study

(Short title: Sibling risk of infection-related hospitalization)

Jessica E. Miller[1,2*], Kim W. Carter[3], Nicholas de Klerk[3], David P. Burgner[1,2,4]

Affiliations:

[1] Murdoch Children's Research Institute, Royal Children's Hospital, Parkville, Victoria, Australia

[2] Department of Paediatrics, University of Melbourne, Parkville, Victoria, Australia

[3] Telethon Kids Institute, University of Western Australia, Perth, Western Australia, Australia

[4] Department of Paediatrics, Monash University, Clayton, Victoria, Australia

***** Corresponding author

Email: jessica.miller@mcri.edu.au (JEM)

Abstract

Objective: To assess the risk of severe childhood infections within families, we conducted a sibling analysis in a population-based cohort study with genealogical linkage. We investigated the sibling risk of hospitalization with common infections, a marker of severity. We hypothesized that having siblings hospitalized for infection would increase the proband's subsequent risk of admission with infection.

Study design: We used population data on Western Australian live-born singletons and their siblings between 1980 and 2014. Measures of infection were infection-related hospitalizations from discharge diagnostic codes. Exposure was having a sibling who had an infection-related hospitalization. Outcomes were infection-related hospitalizations in the child/proband. Probands were followed until an infection-related hospitalization admission (up to the first three), death, 18th birthday, or end of 2014, whichever occurred first. Infection risks were estimated by adjusted Cox proportional hazard models for multiple events.

Results: Of 512,279 probands, 142,915 (27.9%) had infection-related hospitalizations; 133,322 (26.0%) had a sibling with a previous infection-related hospitalization (i.e. exposed). Median interval between sibling and proband infection-related hospitalizations was 1.4 years (inter-quartile range 0.5-3.7). Probands had a dose-dependent increase in risk if sibling/s had 1, 2, or 3+ infection-related hospitalizations (adjusted hazard ratio, aHR 1.41, 95% CI 1.39-1.43; aHR 1.65, 1.61-1.69; aHR 1.83, 1.77-1.90, respectively). Among siblings with the same clinical infection type, highest sibling risks were for genitourinary (aHR 2.06, 1.68-2.53), gastrointestinal (aHR 2.07, 1.94-2.19), and skin/soft tissue infections (aHR 2.34, 2.15-2.54). Overall risk of infection-related hospitalization was higher in children with more siblings and with older siblings.

Conclusion: In this population-based study, we observed an increased risk of infection-related hospitalization in children whose siblings were previously hospitalized for infection. Public health interventions may be particularly relevant in families of children hospitalized with infection.

Abbreviations: Western Australia (WA); Socio-economic status (SES); Hazard ratio (HR); Confidence interval (CI); Interquartile range (IQR)

Introduction

Infection is a predominant cause of childhood morbidity, mortality and health service utilization. US national estimates from 2000-2012 indicate that infections in children aged <19 years accounted for 24.5% of all hospitalizations.[1] In 2003, 43% of US children aged <1 year admitted to hospital had an infection-related diagnosis, incurring a total cost of $690 million.[2]

Microbial factors, such as outbreaks and heightened pathogen virulence, partly explain the marked variation in infection severity following exposure to similar pathogens.[3] Sibling analyses can account for shared heritable and environmental factors and measure associations from exposures at different ages in siblings.[4] Sibling analyses provide robust estimates of shared heritable determinants of infection that do not reflect seasonal variation in pathogen epidemiology and virulence. In genetic epidemiology, sibling risk ratio, or relative recurrence risk, is the ratio of risk in a sibling to a general population risk. Few studies have estimated the sibling risk ratio of infectious diseases.[5]

We utilized total population data from Western Australia (WA) to estimate the risk of hospitalizations for childhood infections within families. We hypothesized that having siblings hospitalized for infection would increase the proband's subsequent risk of admission with infection. We explored whether susceptibility varied by specific types of infections, the age and number of siblings, and the interval between sibling and proband infection-related hospitalizations.

Methods

Study population

Since 2011, WA has had an average growth rate of 1.81% and currently, has a population of ~2.6 million people.[6] We conducted a population-based cohort study of live-born children in WA between January 1st 1980 and December 31st 2014, identified from the WA Birth Registry. Data from the WA Midwives' Notification System, Birth Register, Death Register, and Hospital Morbidity Database System were linked by probabilistic matching at the Data Linkage Branch of the WA Department of Health. Siblings were identified and their data linked with the Family Connections System, which creates links for pedigree relationships based on birth, death and marriage registrations.[7] Single child families and families with twins or higher multiples were excluded from the study; multiples may share virulent pathogens causing more severe contemporaneous infection and genetically identical multiples might inflate heritability estimates derived from siblings alone.[8] Half-siblings were not included as it was unknown when they became siblings or whether they resided in the same household. Individuals of Aboriginal or Torres Strait Islander descent were not included, as causal pathways are likely to be qualitatively different and include substantial economic disadvantage.[9]

The study was approved by the Department of Health Human Research Ethics Committee (HREC), Murdoch Children's Research HREC and University of WA HREC.

Infection-related hospitalization (exposure and outcome)

Data on infection-related hospitalizations were obtained from the Hospital Morbidity Database System, which has complete coverage of private and public WA hospital admissions.[10]

Discharge diagnostic codes are based on the International Classification of Diseases (ICD) versions 9 and 10-AM and have been repeatedly validated.[10]

Infection-related hospitalization is a robust and widely accepted measure of infection severity and reflects hospital admissions due to infections of sufficient severity to warrant inpatient care.[9,11] Infection-related hospitalizations were identified using principal and up to twenty additional ICD codes, a more sensitive measure of total infection burden than the principal code alone that has been used by other researchers for similar purposes.[9,12] An infection-related hospitalization was defined as admission with ≥1 infection discharge code occurring ≥24 hours after discharge for the birth-related hospitalization. We restricted infection-related hospitalizations to readmission following the birth-related discharge to avoid bias from suspected neonatal sepsis that is often either unconfirmed or caused by direct microbial exposure during birth. Overlapping or nested hospital transfers, where <24 hours had elapsed between discharge and re-hospitalization, and repeat infection-related hospitalizations within 7 days were considered as single admissions.

ICD infection codes were categorized *a priori* into seven broad clinical groups: invasive bacterial, gastrointestinal, lower respiratory tract, upper respiratory tract, skin and soft tissue, genitourinary, and viral infections, as previously described.[13] Additionally, we assessed specific common infections: tonsillitis (acute and chronic), otitis media (chronic), gastroenteritis and bronchiolitis; acute otitis media was uncommon and not included.

Exposure was defined as infection-related hospitalizations occurring in a sibling (‘sibling infection-related hospitalization’), categorized as (0, 1, 2, 3+). The child of analysis, termed ‘proband’ hereafter, became ‘exposed’ once a sibling had an infection-related hospitalization during the follow-up period. Exposure was considered time-varying; once a proband became

exposed, they were exposed for the remainder of the follow-up period. Probands were 'unexposed' for the period preceding the first sibling infection-related hospitalization or if no sibling infection-related hospitalizations occurred during follow-up. The study outcome was infection-related hospitalizations, up to the first 3 admissions, occurring in the proband during the follow-up period.

Covariates

Data on maternal age, smoking during pregnancy, parity, birth weight, gestational age, sex, Apgar score, and birth mode were obtained from the Midwives' Notification System, which collates details on all births in WA from 1980 onwards. Percentiles for area-level socioeconomic status (SES) were defined by matching address at birth to average socioeconomic census data for the same census Collector's District from the census year closest to the birth year.[14] Data on smoking during pregnancy were available from 1997 onwards.

Statistical methods

We estimated sibling risk ratios as the risk of infection-related hospitalizations in exposed probands (i.e. those whose sibling/s had an infection-related hospitalization) compared to the risk in unexposed probands (i.e. those whose sibling/s had not had infection-related hospitalization/s). For all children, follow-up time began when they became a sibling: for children with older siblings, follow-up time began at the child's birth-related hospital discharge date; for children with only younger siblings, follow-up time began at the date of birth of their first sibling. All children were followed until they had an infection-related hospitalization admission (up to the first three admissions), death, 18th birthday, or end of 2014, whichever occurred first. Multivariable analyses adjusted for maternal age (<20, 20-24, 25-29, 30-34, ≥35 years), parity

(previous births no/yes), gestational age (<28, 28-29, 30-31, 32-34, 35, 36, 37, 38, 39-40, 41, ≥42 weeks), birth weight (1000-1499, 1500-1999, 2000-2499, 2500-2999, 3000-3499, 3500-3999, ≥4000 grams), sex, season of birth (spring, summer, autumn, winter), 5-minute Apgar score (0-7, 8-10), birth mode (vaginal/cesarean), and year (2 year blocks). Adjusted hazard ratios (HRs) and 95% confidence intervals (CIs) were estimated using multivariate Cox proportional hazard regression models for recurrent events data (conditional risk set models) by Prentice et al.[15] Proband infection-related hospitalizations were the events and age in months was the underlying time variable. Time to each event was measured from entry time, and analyses were stratified by event order. Children in a family generally contributed to the analyses both as a proband and as a sibling, when their sibling was the proband. Depending on family size, children could occur in more than one proband/sibling unit. Robust standard errors accounted for dependence of family members.

Data on smoking during pregnancy (no/yes) were not included in overall analyses, as these data were unavailable for the entire study period. Given the missing values for smoking and SES, sensitivity analyses were conducted including and excluding these variables. All other covariates had few missing data; no imputations were warranted.

We estimated sibling risk ratios for overall, clinical group, and specific common infections. For clinical and specific infection sibling risk ratios, risks were estimated among probands and siblings who presented with the same clinical group or specific infection. We additionally estimated clinical group estimates if siblings presented with any infection. For specific infections, we conducted stratified analyses for siblings younger or older than the proband. For simplicity and to explore an age effect, we restricted these analyses to families where siblings were either all younger or all older than the proband.

We calculated overall infection-related hospitalization risk in children age <5 and 5-18 years by the time-at-risk for infection-related hospitalization following exposure. Time-at-risk was defined among the unexposed as the time from the start of follow-up to the first proband infection-related hospitalization or end of study, whichever occurred first, and among the exposed as the time from the first sibling infection-related hospitalization to the first proband infection-related hospitalization or end of study, whichever occurred first. Within time-at-risk intervals and exposure group, infection-related hospitalization risk was calculated as number of cases divided by total number unexposed or exposed, respectively.

We estimated sibling risk ratios by family size (1, 2, 3+ siblings) and by age of siblings (younger/older than proband), accounting for the number and distribution of the sibling infection-related hospitalizations. Analyses by age of sibling were restricted to families where all siblings were either younger or older than the proband. To assess the possible contribution of pathogen sharing, we calculated the time between admission in the sibling and first admission in the proband with the same clinical infection type. To minimize risk due to pathogen sharing among siblings, we also performed analyses excluding those whose proband infection-related hospitalization admissions occurred within 30 days of the sibling exposure.

We performed a sensitivity analysis using infection-related hospitalizations identified by the principal and first 3 additional ICD codes. To address potential unmeasured confounding, we calculated an E-value, which estimates the minimum strength of association that an unmeasured confounder would need to have with both the exposure and outcome in order to fully explain away the observed association.[16] Lastly, we calculated population attributable fractions for all infections and for bronchiolitis. Analyses were performed in STATA 13.0.[17]

Results

Of the 512,279 probands, 142,915 (27.9%) had at least one infection-related hospitalization and 49,582 (9.7%) had multiple infection-related hospitalization, with a total of 212,882 proband infection-related hospitalizations during follow-up. By the end of follow-up, 133,322 probands (26.0%) were exposed, i.e. had a sibling with an infection-related hospitalization. On average, exposed probands were first-born children with younger mothers (Table 1). The median age of when probands were exposed was 4.7 years (interquartile range, IQR 2.7-7.7) among those who did not have an infection-related hospitalization during follow-up, and 4.2 (IQR 2.2-7.1) among those who did. The median age in probands for their first infection-related hospitalization was 3.2 years (IQR 1.2-6.0); among exposed 5.5 years (IQR 3.0-9.1) and among unexposed 2.8 (IQR 1.1-5.4). The median time between a sibling and proband infection-related hospitalization was 1.4 years (IQR 0.5-3.7).

Table 1 – Characteristics of proband children in study population, time-varying exposure status at end of follow-up period

	Infection-related hospitalization in sibling(s) (Exposure)						
	No (Unexposed)		Yes (Exposed)		Total		Chi-squared
	N	%	N	%	N	%	p-value*
**Characteristic**	378,957	74	133,322	26	512,279	100	
**Maternal age at birth**							
<20	10,619	2.8	6,151	4.6	16770	3.27	0.000
20-24	66,727	17.6	31,177	23.4	97904	19.11	

25-29	131,256	34.6	49,307	37.0	180563	35.25	
30-34	117,912	31.1	34,798	26.1	152710	29.81	
≥35	52,440	13.8	11,888	8.9	64328	12.56	
Missing	3	0.0	1	0.0	4	0.0	
**Maternal parity (had previous birth(s))**							
No	141,861	37.4	61,083	45.8	202,944	39.6	0.000
Yes	237,096	62.6	72,239	54.2	309,335	60.4	
Missing	0	0.0	0	0.0	0	0.0	
**Sex**							
Male	197,026	52.0	66,469	49.9	263,495	51.4	0.000
Female	181,927	48.0	66,853	50.1	248,780	48.6	
Missing	4	0.0	0	0.0	4	0.0	
**Season of birth**							
Spring	94,813	25.0	33,223	24.9	128,036	25.0	0.707
Summer	91,339	24.1	32,245	24.2	123,584	24.1	
Autumn	97,757	25.8	34,276	25.7	132,033	25.8	
Winter	95,048	25.1	33,578	25.2	128,626	25.1	
Missing	0	0.0	0	0.0	0	0.0	
**Apgar score 5 minutes**							
0-7	9,899	2.6	3,597	2.7	13,496	2.6	0.146
8-10	368,796	97.3	129,644	97.2	498,440	97.3	
Missing	262	0.1	81	0.1	343	0.0	
**Birth mode**							
Vaginal	283,643	74.9	102,430	76.8	386,073	75.4	0.000
Cesarean	95,314	25.2	30,892	23.2	126,206	24.6	

Missing	0	0.0	0	0.0	0	0.0	
**Birth year**							
1980-84	30,956	8.2	13,528	10.2	44,484	8.7	0.000
1985-89	51,432	13.6	22,209	16.7	73,641	14.4	
1990-94	56,531	14.9	24,588	18.4	81,119	15.8	
1995-99	57,653	15.2	24,155	18.1	81,808	16.0	
2000-04	58,840	15.5	21,452	16.1	80,292	15.7	
2005-10	87,209	23.0	22,934	17.2	110,143	21.5	
2011-13	36,336	9.6	4,456	3.3	40,792	8.0	
Missing	0	0.0	0	0.0	0	0.0	
**Gestational age (weeks)**							
<28	533	0.1	135	0.1	668	0.1	0.000
28-29	592	0.2	187	0.1	779	0.2	
30-31	969	0.3	319	0.2	1,288	0.3	
32-34	4,635	1.2	1,615	1.2	6,250	1.2	
35	4,461	1.2	1,703	1.3	6,164	1.2	
36	9,641	2.5	3,473	2.6	13,114	2.6	
37	28,210	7.4	9,892	7.4	38,102	7.4	
38	81,052	21.4	26,706	20.0	107,758	21.0	
39-40	195,890	51.7	69,037	51.8	264,927	51.7	
41	47,410	12.5	17,814	13.4	65,224	12.7	
≥42	5,145	1.4	2,232	1.7	7,377	1.4	
Missing	419	0.1	209	0.2	628	0.1	
**Birthweight (grams)**							
<1000	564	0.2	141	0.1	705	0.1	0.000

1000-<1500	1,120	0.3	384	0.3	1,504	0.3	
1500-<2000	2,342	0.6	814	0.6	3,156	0.6	
2000-<2500	9,605	2.5	3,575	2.7	13,180	2.6	
2500-<3000	53,240	14.1	19,384	14.5	72,624	14.2	
3000-<3500	144,371	38.1	51,146	38.4	195,517	38.2	
3500-<4000	123,930	32.7	43,106	32.3	167,036	32.6	
≥4000	43,779	11.6	14,769	11.1	58,548	11.4	
Missing	6	0.0	3	0.0	9	0.0	
**Maternal socioeconomic status (percentile)**							
lowest <10	23,606	6.2	10,071	7.6	33,677	6.6	0.000
10-25	46,252	12.2	17,538	13.2	63,790	12.5	
25-50	83,934	22.2	30,553	22.9	114,487	22.4	
50-75	88,923	23.5	29,572	22.2	118,495	23.1	
75-90	61,186	16.2	19,125	14.3	80,311	15.7	
highest >90	42,552	11.2	12,881	9.7	55,433	10.8	
Missing	32,504	8.6	13,582	10.2	46,086	9.0	
**Maternal smoking during pregnancy**							
No	184,327	48.6	51,065	38.3	235,392	46.0	0.000
Yes	24,816	6.6	8,610	6.5	33,426	6.5	
Missing	169,814	44.8	73,647	55.2	243,461	47.5	

*Chi-square test of independence for exposure status and dichotomous and categorized measures

We observed an increased risk of infection-related hospitalization among probands whose sibling/s had infection-related hospitalization/s. The risk increased in a dose-dependent manner

with number of infection-related hospitalizations in the sibling/s (1, 2, 3+) (adjusted hazard ratio, aHR 1.41, 95% CI 1.39-1.43; aHR 1.65, 1.61-1.69; aHR 1.83, 1.77-1.90, respectively) (Fig 2A).

Among families where the proband and their sibling were hospitalized with the same clinical infection type, the median time between sibling and proband hospitalizations ranged from 0.7 years (IQR 0.01-2.9) for gastrointestinal infections, to 2.9 (IQR 1.5-5.9) for invasive bacterial infections. The highest risks were for genitourinary (aHR 2.06, 1.68-2.53), gastrointestinal (aHR 2.07, 1.94-2.19) and skin and soft tissue infections (aHR 2.34, 2.15-2.54). Risks were lower for any infection than for the same clinical infection group (Table 2).

Table 2 - Infection-related hospitalization sibling hazard ratios by clinical infection groups

Infection-related hospitalization sibling hazard ratios by type of clinical infection in proband (outcome) and sibling (exposure), compared to risk in probands whose sibling/s did not have infection-related hospitalizations (reference group).

Type of infection in proband	Type of infection in sibling	Number of infections in sibling	Adjusted HR (95% CI)
**Genitourinary**			
	Any infection	Yes	1.42 (1.33-1.51)
	Genitourinary	Yes	2.06 (1.68-2.53)
		1	1.99 (1.61-2.45)
		2+	3.23 (1.61-6.46)
**Invasive bacterial**			
	Any infection	Yes	1.3 (1.19-1.42)
	Invasive bacterial	Yes	1.44 (0.87-2.38)

		1	1.42 (0.85-2.38)
		2+	2.21 (0.33-14.97)
**Lower respiratory tract**			
	Any infection	Yes	1.41 (1.37-1.46)
	Lower respiratory tract	Yes	1.69 (1.61-1.77)
		1	1.65 (1.57-1.73)
		2+	2.12 (1.84-2.45)
**Upper respiratory tract**			
	Any infection	Yes	1.45 (1.43-1.47)
	Upper respiratory tract	Yes	1.64 (1.61-1.67)
		1	1.61 (1.58-1.64)
		2+	1.80 (1.74-1.87)
**Viral**			
	Any infection	Yes	1.40 (1.35-1.44)
	Viral	Yes	1.76 (1.64-1.88)
		1	1.72 (1.60-1.85)
		2+	2.37 (1.84-3.05)
**Gastrointestinal**			
	Any infection	Yes	1.48 (1.43-1.54)
	Gastrointestinal	Yes	2.07 (1.95-2.20)
		1	2.04 (1.92-2.18)

		2+	2.43 (1.98-2.97)
**Skin and soft tissue**			
	Any infection	Yes	1.46 (1.4-1.52)
	Skin and soft tissue	Yes	2.34 (2.16-2.54)
		1	2.33 (2.14-2.53)
		2+	2.52 (1.89-3.36)

Adjusted for maternal age (<20, 20-24, 25-29, 30-34, ≥35 years), parity (previous births no/yes), gestational age (<28, 28-29, 30-31, 32-34, 35, 36, 37, 38, 39-40, 41, ≥42 weeks), birth weight (1000-1499, 1500-1999, 2000-2499, 2500-2999, 3000-3499, 3500-3999, ≥4000 grams), sex of the child, season of birth (spring, summer, autumn, winter), 5-minute Apgar score (0-7, 8-10), birth mode (vaginal/cesarean), birth year (2 year blocks).

Number of infections in sibling was analyzed as both dichotomous (Yes/No (reference)) or categorical (0 (reference), 1, 2+).

For the specific infections, the median time between sibling and proband infection-related hospitalization ranged from 40 days for bronchiolitis to 2.4 years for chronic tonsillitis. The highest observed risk was for chronic tonsillitis among probands who had an older sibling previously hospitalized for chronic tonsillitis (aHR 3.08, 2.92-3.24) (S1 Fig).

Overall risks of infection were highest among preschool children (age <5 years) (Fig 3). For all children, the highest risk for an infection-related hospitalization occurred among children whose sibling was hospitalized with an infection within the previous month (0.93 in exposed children <5 years, 0.63 in exposed children 5-18 years). In children <5 years, the risk difference between exposed and unexposed children remained fairly constant regardless of when, in the 12 months

prior to a proband infection-related hospitalization, the exposure occurred. In children aged 5-18 years, the risk difference between exposed and unexposed was similar regardless of whether exposure occurred one month to 2 years prior to a proband infection-related hospitalization.

The dose-response effect with increasing number of sibling infection-related hospitalizations persisted despite family size. However, with increasing family size, overall infection-related hospitalization risks slightly decreased. Overall risk of infection was slightly higher in children with older siblings (Fig 4A). Increased infection-related hospitalization risk among exposed probands persisted even when proband infection-related hospitalization occurring within 30 days of a sibling infection-related hospitalization were excluded (Fig 4B). In families with 2+ siblings, we did not observe any pattern of association with sibling infections occurring in one or multiple siblings (Fig 4C).

Sensitivity analyses including only infections coded as the principal or first three additional diagnostic codes showed similar results (S1 Table). Based on the E-value, an unmeasured confounder would need an association of ≥1.85 with infection-related hospitalization in both the sibling and proband, to explain away the observed associations, but weaker confounding could not.

We calculated that 12.2% (11.8-12.6) (n=29027) of infection-related hospitalizations in the population would be potentially prevented if siblings did not have prior infection-related hospitalizations. If multiple infection-related hospitalizations in siblings were reduced, 4.4% (4.2-4.6) (n=10,469) of infection-related hospitalizations in probands could be prevented. 1.3% (0.5-2.1) (n=200) of infection-related hospitalizations for bronchiolitis would be prevented if siblings did not have bronchiolitis.

Discussion

The risk of hospitalization with infection was increased among children whose siblings had previous infection-related hospitalizations, compared with children whose siblings had no previous infection-related hospitalizations. The risk increased in a dose-response manner from 40% in children with one sibling infection-related hospitalization to 80% with ≥3 sibling infection-related hospitalizations.

Increased risks were observed for all clinical and specific infection groups. The long median interval (1.4 years) between sibling and proband infection-related hospitalization suggests that factors other than sharing of virulent pathogens or seasonal outbreaks explain the increased risks.[8] Further, when sibling and proband had infection-related hospitalizations in the same clinical group, risks increased, suggesting shared underlying risks, such as more specific heritable and environmental factors.[3,18]

Sibling infection-related hospitalization risk was reduced in larger families, but the mechanisms are unclear. This may reflect differential use of child care, a well-recognized risk factor for childhood infection;[19] families with more older children use less child care. Almost 50% of Australian families with ≤2 children are likely to be enrolled in child care compared to 33% of larger families (3+ children),[20] however, data on child care attendance in our population were unavailable. In addition, larger sibship size is protective against asthma, allergy and some autoimmune diseases in children and adults.[21,22] Early microbial exposures associated with increased family size, such as increased microbiome diversity, may optimise early life immune

responses.[23] Overall risks were slightly lower in families with siblings younger than the proband, likely reflecting reduced susceptibility to more severe infection with increasing age.

The strengths of our study include 35 years of total population linked data with minimal loss to follow-up. The WA Data Linkage System is a powerful and unique resource of data from a stable population.[24] All WA hospital admission data are captured and almost all maternal full siblings have been identified. The Midwives' Notification Form records >99% of all live births.[25] Few other studies have examined sibling risks of infectious diseases apart from those reporting on single infectious diseases.[5]

Our outcome measure of infection-related hospitalization is a marker of infectious disease severity. Hospitalization is less influenced by health-seeking behavior, social disadvantage and physician management than emergency department or primary care presentations and by practitioner-related variation in management [26,27]; further studies using these less severe outcomes of infection are of interest, however these data were unavailable for this study. Inclusion of principal and additional diagnostic codes, an approach used in analogous studies,[12] captures infection burden more completely than the principal code alone. Sensitivity analyses that captured infections based on the principal and first three additional codes showed similar results as when all 20 additional codes were considered.

Sibling studies are more powerful than twin studies for less common but clinically important infections.[3] Infections in sibling pairs (unlike twins) generally occur many months apart, much longer than the incubation period for almost all childhood pathogens. This reduces possible biases from over-diagnosis in the second child, from clinically unwarranted hospitalization with the same infection in the second child (e.g. social factors or parental anxiety), and from the confounding effects of shared virulent pathogens. Although many infections show increased

concordance rates in monozygous versus dizygous twins,[18,28] twin infection studies may overestimate genetic factors and underestimate shared environmental exposures. For example, twin data on tuberculosis, where increased concordance in monozygous twins has long been suggestive of host genetic susceptibility, have been re-analyzed and the findings are suggested to be largely due to environmental factors which may outweigh the importance of hereditary factors.[29]

Population analyses have some unavoidable limitations. Data are limited to routinely collected and recorded information. Data on infections managed in primary care or in emergency departments and data on potential unmeasured co-variates, (e.g. child care attendance, breastfeeding, tobacco smoke exposure, obesity, parental chronic disease, and environmental exposures) were unavailable. Smoking data were available from 1997 onwards; SES data were missing for 9% of the population. We therefore present our main findings with and without adjustments for smoking and SES. We could not assess migration from WA or transfer to facilities outside the state; both are rare.[30] Family Connections uses registrations where parents may not be biological (e.g. adoptions, surrogacy), but these families could not be identified. The estimated small percentage of adoptions is unlikely to skew our results but should be considered when interpreting heritability.

Sibling risks reflect shared genomic susceptibility and environmental exposures. However, the long interval between sibling and proband admission suggests infection within families with virulent organisms is an unlikely explanation for increased risks, especially as most pediatric pathogens are widely distributed and carried asymptomatically in the majority.[3] In a study of meningococcal disease, the sibling risk ratio was 30.3 overall and 8.2 if cases occurred >1 year apart,[8] indicating that a more accurate estimate of the contribution of shared heritable and

environmental factors, independent of pathogen sharing, is likely if the interval between cases is longer than incubation period and asymptomatic carriage of the invasive strain. In our study, we performed a sub-group analysis excluding cases within 30 days following the sibling's infection and the increased risk in the proband remained.

Conclusions

These data help establish the heritable association of hospitalization for common childhood infections among siblings. The findings highlight the importance of preventative measures and efforts to reduce environmental risk factors, especially in families of children hospitalized with infection.

Acknowledgements

The authors would like to thank the staff at the Western Australia Data Linkage Branch. We thank the data custodians of the Birth Registrations, Death Registrations, Hospital Morbidity Data Collection and Midwives Notification System.

Author contributions

All authors contributed to the planning and design of the epidemiological study. JEM and NdK reviewed the raw data and were directly involved in the analysis. DPB, KWC, and NdK provided analytical feedback based on aggregated results. JEM and DPB drafted the manuscript, with input from all authors. All authors provided substantive review and commentary on multiple

drafts and approved the final version. JEM had full access to all of the data in the study and takes responsibility for the integrity of the data and the accuracy of the data analysis.

References

1. Goto T, Tsugawa Y, Mansbach JM, Camargo CA, Jr., Hasegawa K. Trends in Infectious Disease Hospitalizations in US Children, 2000 to 2012. Pediatr Infect Dis J. 2016;35(6):e158-63. Epub 2016/03/12. doi: 10.1097/INF.0000000000001134. PubMed PMID: 26967815; PubMed Central PMCID: PMCPMC4912127.
2. Yorita KL, Holman RC, Sejvar JJ, Steiner CA, Schonberger LB. Infectious disease hospitalizations among infants in the United States. Pediatrics. 2008;121(2):244-52. Epub 2008/02/05. doi: 121/2/244 [pii]

10.1542/peds.2007-1392. PubMed PMID: 18245414.
3. Burgner D, Jamieson SE, Blackwell JM. Genetic susceptibility to infectious diseases: big is beautiful, but will bigger be even better? Lancet Infect Dis. 2006;6(10):653-63. doi: 10.1016/S1473-3099(06)70601-6. PubMed PMID: 17008174; PubMed Central PMCID: PMCPMC2330096.
4. Song H, Fall K, Fang F, Erlendsdottir H, Lu D, Mataix-Cols D, et al. Stress related disorders and subsequent risk of life threatening infections: population based sibling controlled cohort study. BMJ. 2019;367:l5784. Epub 2019/10/28. doi: 10.1136/bmj.l5784. PubMed PMID: 31645334; PubMed Central PMCID: PMCPMC6812608
5. Rostgaard K, Nielsen TR, Wohlfahrt J, Ullum H, Pedersen O, Erikstrup C, et al. Sibship structure and risk of infectious mononucleosis: a population-based cohort study. Int J Epidemiol. 2014;43(5):1607-14. Epub 2014/12/02. PubMed PMID: 25436250.
6. Australia P. Population of Western Australia 2020 [cited 2020 May 1]. Available from: http://www.population.net.au/population-of-western-australia/.
7. Glasson EJ, de Klerk NH, Bass AJ, Rosman DL, Palmer LJ, Holman CD. Cohort profile: The Western Australian Family Connections Genealogical Project. Int J Epidemiol. 2008;37(1):30-5. Epub 2007/07/06. doi: dym136 [pii]

10.1093/ije/dym136. PubMed PMID: 17611241.
8. Haralambous E, Weiss HA, Radalowicz A, Hibberd ML, Booy R, Levin M. Sibling familial risk ratio of meningococcal disease in UK Caucasians. Epidemiol Infect. 2003;130(3):413-8. PubMed PMID: 12825725.
9. Carville KS, Lehmann D, Hall G, Moore H, Richmond P, de Klerk N, et al. Infection is the major component of the disease burden in aboriginal and non-aboriginal Australian children: a population-based study. Pediatr Infect Dis J. 2007;26(3):210-6. PubMed PMID: 17484216.
10. Holman CD, Bass AJ, Rosman DL, Smith MB, Semmens JB, Glasson EJ, et al. A decade of data linkage in Western Australia: strategic design, applications and benefits of the WA data linkage system. Aust Health Rev. 2008;32(4):766-77. Epub 2008/11/05. doi: ahr_32_4_766 [pii]. PubMed PMID: 18980573.
11. Petersen L, Andersen PK, Sorensen TI. Genetic influences on incidence and case-fatality of infectious disease. PLoS One. 2010;5(5):e10603. doi: 10.1371/journal.pone.0010603. PubMed PMID: 20498716; PubMed Central PMCID: PMCPMC2871036.
12. Tielemans S, de Melker HE, Hahne SJM, Boef AGC, van der Klis FRM, Sanders EAM, et al. Non-specific effects of measles, mumps, and rubella (MMR) vaccination in high income setting: population based cohort study in the Netherlands. BMJ. 2017;358:j3862. doi: 10.1136/bmj.j3862. PubMed PMID: 28855159.

13. Miller JE, Hammond GC, Strunk T, Moore HC, Leonard H, Carter KW, et al. Association of gestational age and growth measures at birth with infection-related admissions to hospital throughout childhood: a population-based, data-linkage study from Western Australia. Lancet Infect Dis. 2016. doi: 10.1016/S1473-3099(16)00150-X. PubMed PMID: 27052469.
14. Pink B. An Introduction to Socio-Economic Indexes for Areas (SEIFA). In: Statistics. ABo, editor. Canberra: Australian Bureau of Statistics.; 2006.
15. Prentice RL, Williams BJ, Peterson AV. On the regression analysis of multivariate failure time data. Biometrika. 1981;68(2):373-9. doi: 10.1093/biomet/68.2.373.
16. VanderWeele TJ, Ding P. Sensitivity Analysis in Observational Research: Introducing the E-Value. Ann Intern Med. 2017;167(4):268-74. doi: 10.7326/M16-2607. PubMed PMID: 28693043.
17. StataCorp. STATA Statistical Software: Release 13. 13.0 ed. College Station, TX:2013.
18. Jepson A. Twin studies for the analysis of heritability of infections diseases. B I Pasteur. 1998;96(2):71-81. PubMed PMID: WOS:000078369700001.
19. Kamper-Jorgensen M, Wohlfahrt J, Simonsen J, Gronbaek M, Benn CS. Population-based study of the impact of childcare attendance on hospitalizations for acute respiratory infections. Pediatrics. 2006;118(4):1439-46. doi: 10.1542/peds.2006-0373. PubMed PMID: 17015534.
20. Statistics ABo. Childhood Education and Care (cat. no. 4402.0). Australia: 2008 June 2008. Report No.
21. von Mutius E. The environmental predictors of allergic disease. J Allergy Clin Immunol. 2000;105(1 Pt 1):9-19. Epub 2000/01/12. PubMed PMID: 10629447.
22. Hughes AM, Lucas RM, McMichael AJ, Dwyer T, Pender MP, van der Mei I, et al. Early-life hygiene-related factors affect risk of central nervous system demyelination and asthma differentially. Clin Exp Immunol. 2013;172(3):466-74. Epub 2013/04/23. doi: 10.1111/cei.12077. PubMed PMID: 23600835; PubMed Central PMCID: PMCPMC3646446.
23. Laursen MF, Zachariassen G, Bahl MI, Bergstrom A, Host A, Michaelsen KF, et al. Having older siblings is associated with gut microbiota development during early childhood. BMC Microbiol. 2015;15:154. doi: 10.1186/s12866-015-0477-6. PubMed PMID: 26231752; PubMed Central PMCID: PMCPMC4522135.
24. Stanley FJ, Croft ML, Gibbins J, Read AW. A population database for maternal and child health research in Western Australia using record linkage. Paediatr Perinat Epidemiol. 1994;8(4):433-47. PubMed PMID: 7870627.
25. Gee V HQ, Ernstzen AN. . Perinatal Statistics in Western Australia, 2005: Twenty third Annual Report of the Western Australian Midwives' Notification System. Department of Health, Western Australia, 2007.
26. Saxena S, Majeed A, Jones M. Socioeconomic differences in childhood consultation rates in general practice in England and Wales: prospective cohort study. BMJ. 1999;318(7184):642-6. PubMed PMID: 10066207; PubMed Central PMCID: PMCPMC27771.
27. Watson RL, Dowell SF, Jayaraman M, Keyserling H, Kolczak M, Schwartz B. Antimicrobial use for pediatric upper respiratory infections: reported practice, actual practice, and parent beliefs. Pediatrics. 1999;104(6):1251-7. PubMed PMID: 10585974.
28. Casselbrant ML, Mandel EM, Fall PA, Rockette HE, Kurs-Lasky M, Bluestone CD, et al. The heritability of otitis media: a twin and triplet study. JAMA. 1999;282(22):2125-30.
29. van der Eijk EA, van de Vosse E, Vandenbroucke JP, van Dissel JT. Heredity versus environment in tuberculosis in twins: the 1950s United Kingdom Prophit Survey Simonds and

Comstock revisited. Am J Respir Crit Care Med. 2007;176(12):1281-8. Epub 2007/09/08. doi: 200703-435OC [pii]

10.1164/rccm.200703-435OC. PubMed PMID: 17823356.
30. Statistics ABo. Migration, Australia 2013-14 2015 [6/11/2015]. Available from: http://www.abs.gov.au/ausstats/abs@.nsf/mf/3412.0/.

Supporting information

S1 Fig. Infection-related hospitalization sibling risk ratios for specific infection groups

Infection-related hospitalization sibling risk ratios among probands whose sibling/s had infection-related hospitalization/s for the same specific infection compared to risk in probands whose siblings/s did not have infection-related hospitalizations (reference group), among younger and older siblings

Adjusted for number of previous infection-related hospitalizations in proband (0, 1, 2+), maternal age (<20, 20-24, 25-29, 30-34, ≥35 years), parity (previous births no/yes), gestational age (<28, 28-29, 30-31, 32-34, 35, 36, 37, 38, 39-40, 41, ≥42 weeks), birth weight (1000-1499, 1500-1999, 2000-2499, 2500-2999, 3000-3499, 3500-3999, ≥4000 grams), sex of the child, season of birth (spring, summer, autumn, winter), 5-minute Apgar score (0-7, 8-10),birth mode (vaginal/cesarean), birth year (2 year blocks).

ICD 9 and 10 codes for bronchiolitis:466.1, J21; gastroenteritis:009, 535.4 535.7, A09; otitis media chronic:381.1, 381.2, 381.3, 381.4, 382.1, 382.2, 382.3, H65.2, H65.3, H65.4, H65.9, H66.1, H66.2, H66.3; tonsillitis acute:034.0, 463, J03; tonsillitis chronic:474, J35. Acute otitis media 381.0, 382.0, H65.0, H65.1, H66.0 was uncommon and not included.

S1 Table. Sensitivity analysis of infection-related hospitalization identification

S2 Fig. Time from exposure (sibling infection-related hospitalization) to outcome (proband infection-related hospitalization) for all and specific infection groups

Figure Legend

Fig 1 – Flowchart of study population

Fig 2A - Overall infection-related hospitalization risk among probands whose sibling/s had infection-related hospitalizations compared to risk in probands whose siblings/s did not have infection-related hospitalizations (reference group). Adjusted for maternal age, parity, gestational age, birth weight, sex, season of birth, 5-minute Apgar score, birth mode, and birth year. Additional adjustments for SES (from Socio-Economic Index for Areas (SEIFA) data) and smoking during pregnancy. Smoking data available from 1997-2013.

Fig 2B - Infection-related hospitalization risks excluding proband infection-related hospitalization admissions occurring within 30 days following a sibling infection-related hospitalization exposure. Adjusted for maternal age, parity, gestational age, birth weight, sex, season of birth, 5-minute Apgar score, birth mode, and birth year. Additional adjustments for SES (from Socio-Economic Index for Areas (SEIFA) data) and smoking during pregnancy. Smoking data available from 1997-2013.

Fig 3 - Risk of 1st occurrence infection-related hospitalization in children <5 and 5-18 years old, by time-at-risk. In the exposed group, 'time-at-risk' is the time from the first sibling infection-related hospitalization (occurring prior to the 1st proband infection-related hospitalization) to the 1st proband infection-related hospitalization or end of study, whichever occurred first. In the unexposed group, 'time-at-risk' begins with the start of follow-up. Follow-up times shorter than the 'time-at-risk' interval were not included in the interval risk calculations. 'Cases' refers to probands with infection-related hospitalizations; 'Non cases' refers to probands with no infection-related hospitalizations.

Fig 4A - Infection-related hospitalization sibling risk ratios by family size and age of siblings.

Adjusted for maternal age, parity, gestational age, birth weight, sex of the child, season of birth, 5-minute Apgar score, birth mode, birth year.

Fig 4B - Infection-related hospitalization sibling risk ratios excluding proband infection-related hospitalization admissions occurring within 30 days following a sibling infection-related hospitalization exposure. Adjusted for maternal age, parity, gestational age, birth weight, sex of the child, season of birth, 5-minute Apgar score, birth mode, birth year.

Fig 4C – Infection-related hospitalization sibling risk ratio by family size and distribution of infections among siblings. Adjusted for maternal age, parity, gestational age, birth weight, sex of the child, season of birth, 5-minute Apgar score, birth mode, birth year. IRH = infection-related hospitalization.

Figure 1
Click here to access/download;Figure;Fig1.tif

Fig 1 – Flowchart of study population

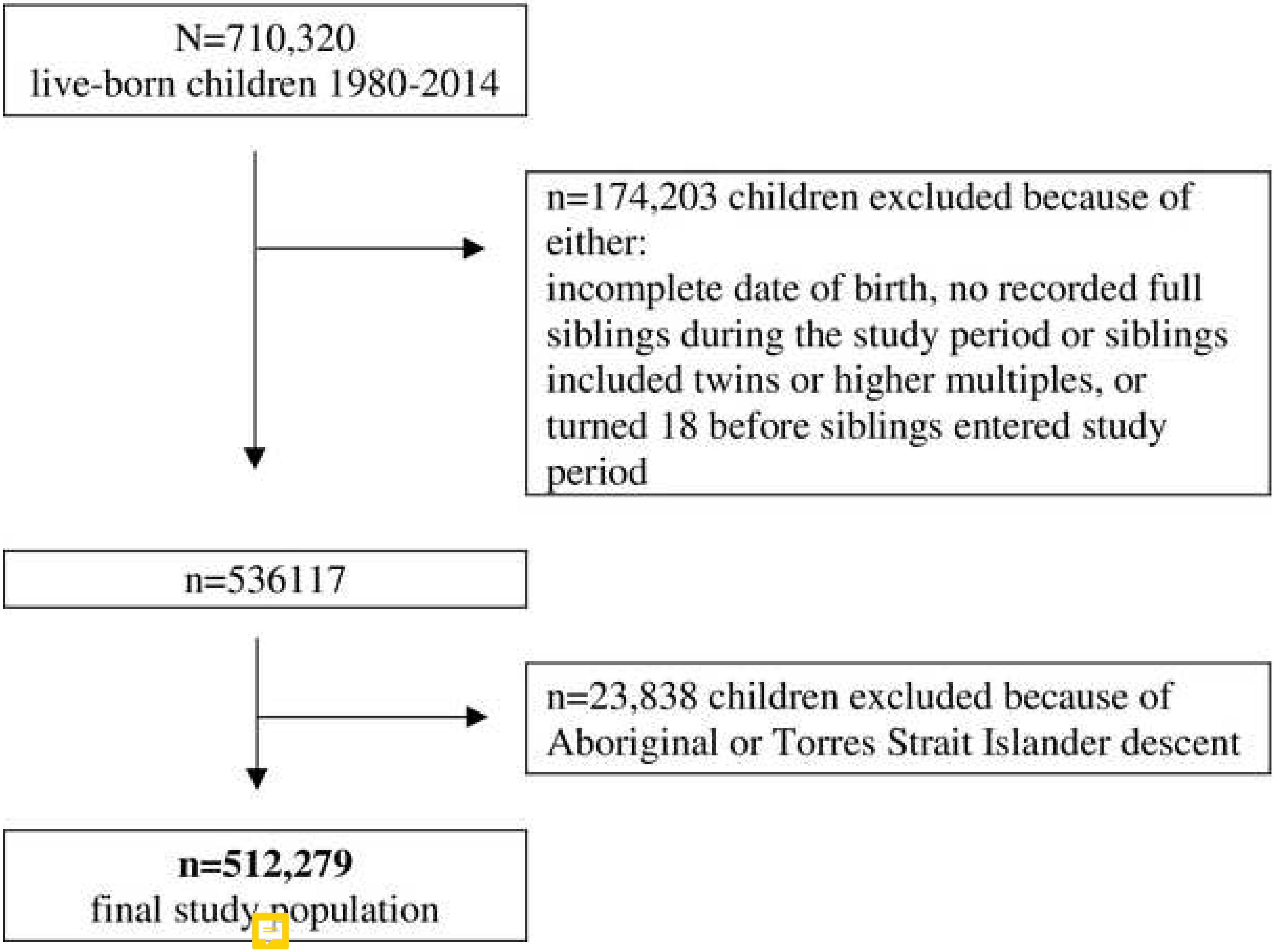

Figure 2A

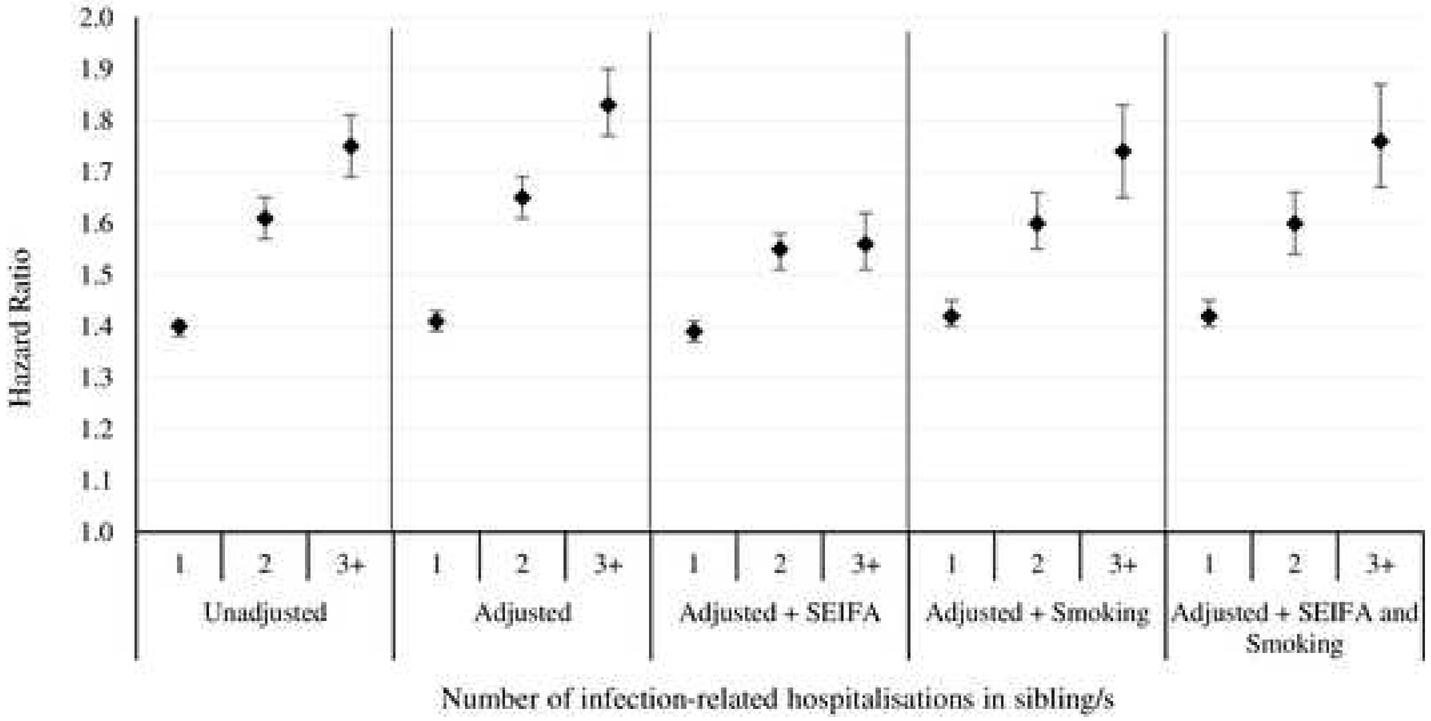

Figure 2B

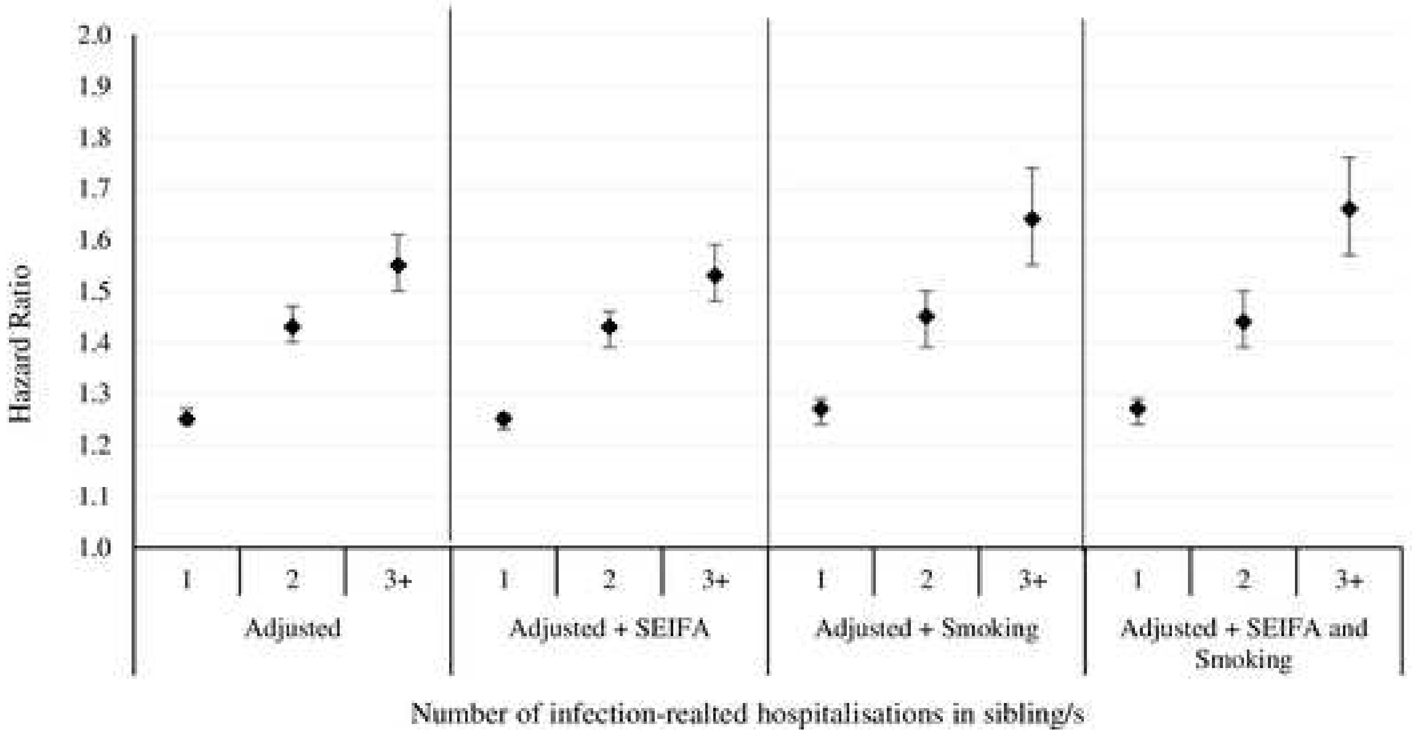

Figure 3

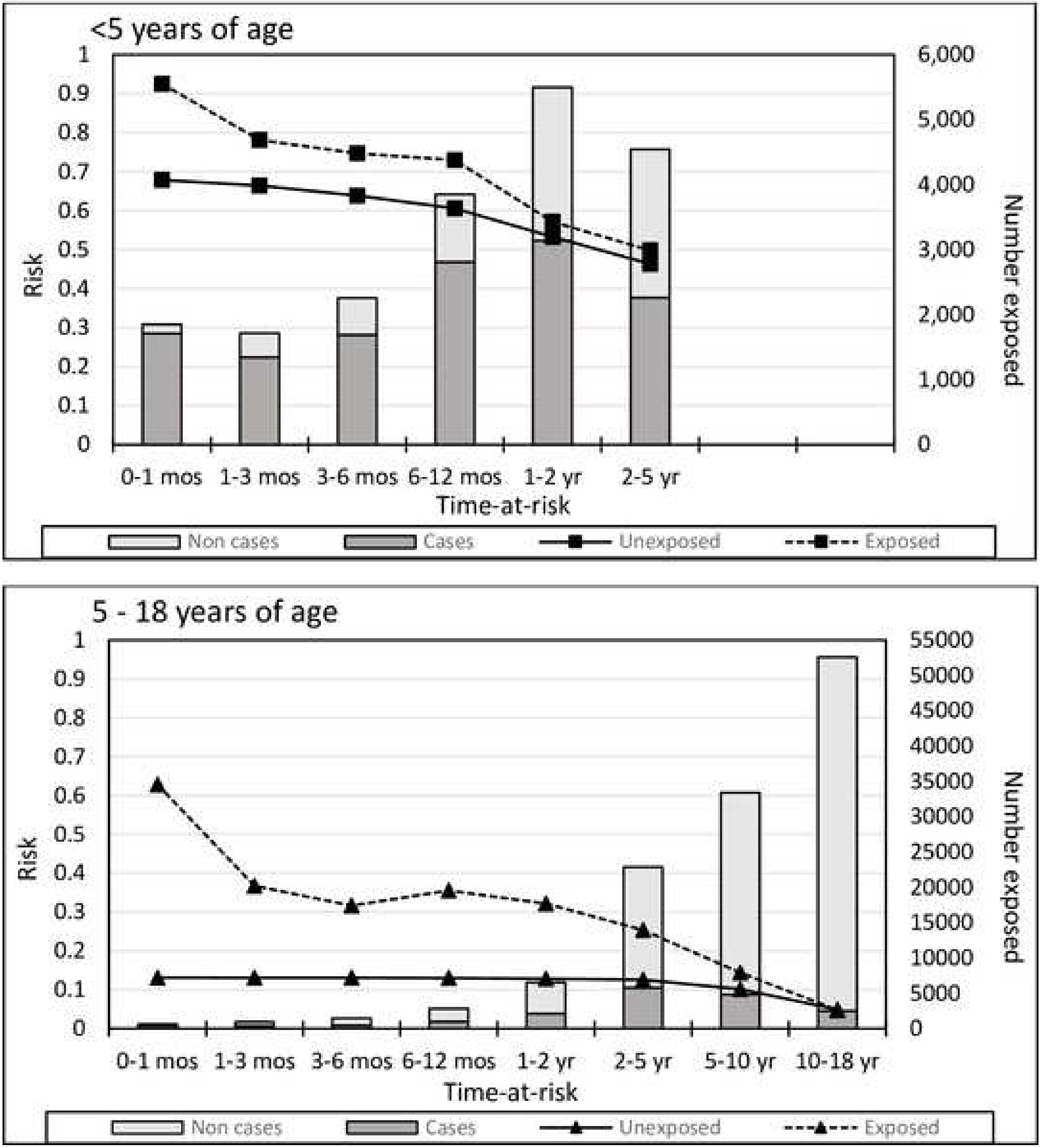

<5 years of age
Risk
Number exposed
0-1 mos
1-3 mos
3-6 mos
6-12 mos
1-2 yr
2-5 yr
Time-at-risk
Non cases
Cases
Unexposed
Exposed
5 - 18 years of age
Risk
Number exposed
0-1 mos
1-3 mos
3-6 mos
6-12 mos
1-2 yr
2-5 yr
5-10 yr
10-18 yr
Time-at-risk
Non cases
Cases
Unexposed
Exposed

Figure 4

Click here to access/download;Figure;Fig4.tif

Figure 4A

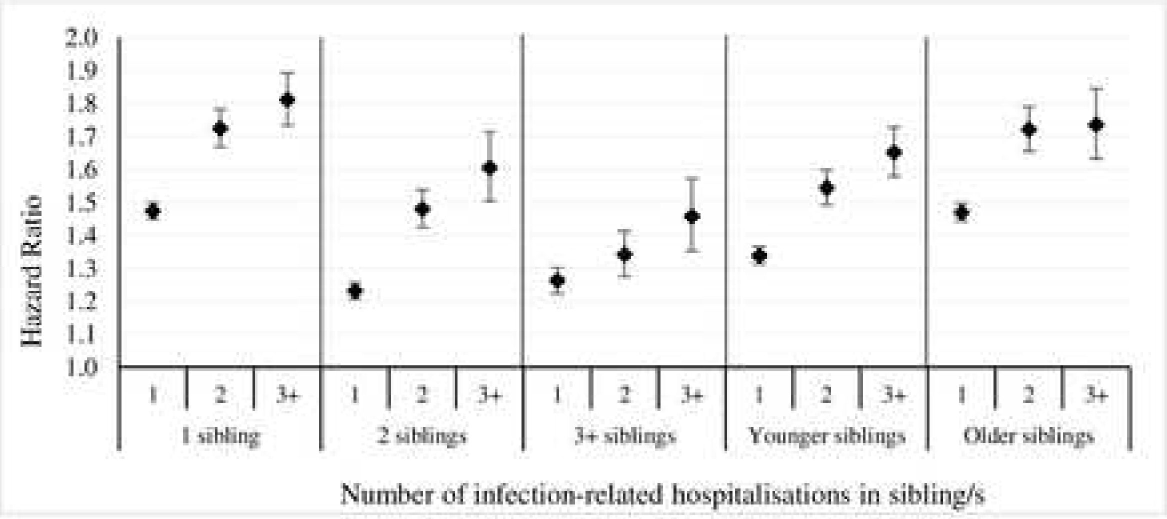

Figure 4B

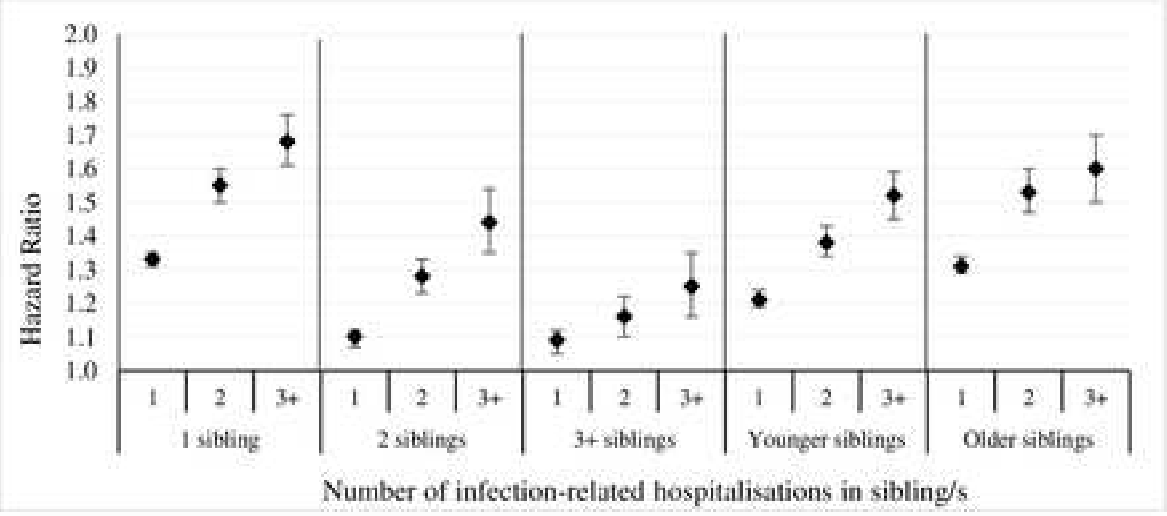

Figure 4C

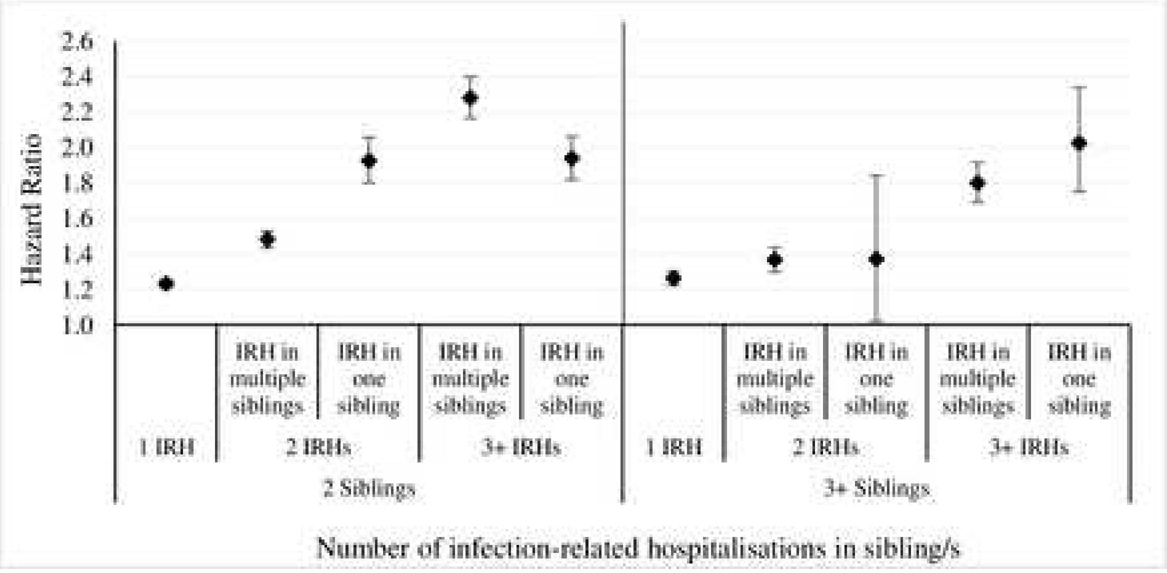

S1 Fig

Click here to access/download

Supporting Information

S1_Fig.pdf

S1 Table

Click here to access/download
Supporting Information
S1_Table.pdf

S2 Fig

Click here to access/download
Supporting Information
S2_Fig.pdf

Click here to access/download
Supporting Information
STROBE_checklist_cohort.docx
